# Periodontitis, risk factors, and their impact on oral health-related quality of life (OHRQOL) among Egyptian Geriatric patients: a multi-center cross-sectional study

**DOI:** 10.1186/s12903-025-06376-6

**Published:** 2025-07-10

**Authors:** Suzan Seif Allah Ibrahim, Mohammed Al-Bahrawy, Aya Hosny Taha Zahran

**Affiliations:** 1 Periodontology and Oral Diagnosis, Faculty of Dentistry, Ain Shams University, Cairo, Egypt; 2 Periodontology and Oral Diagnosis Faculty of Dentistry - Ain Shams University, Cairo, Egypt; 3 Ain Shams University, Cairo, Egypt; 4 Periodontology and Oral Diagnosis Faculty of Oral and Dental Medicine, Nahda University, Beni Suef, Egypt

**Keywords:** Periodontitis, Risk factors, Geriatrics, Oral health-related quality of life OHRQOL

## Abstract

**Aim:**

This study investigated the prevalence, risk indicators, and impact of periodontitis on oral health-related quality of life (OHRQOL) among Egyptian geriatric patients. This study aims to provide insights into the broader implications of this disease for quality of life by assessing functional, psychological, and social domains.

**Methods:**

A cross-sectional study was conducted among 400 Egyptian participants aged 60 years and older recruited from Ain Shams University's outpatient clinics and the Ministry of Health dental research centers. Sociodemographic and behavioral data were collected via structured questionnaires, whereas OHRQOL was evaluated using the OHIP-14 tool. Clinical periodontal assessments adhered to the 2017 World Workshop classification. Logistic regression was applied to identify associations between periodontitis severity, risk indicators, and OHRQOL impacts.

**Results:**

Among the participants, 66.5% (*N* = 266) reported a negative impact on OHRQOL, predominantly due to psychological discomfort, physical pain, and disability. Stages II (40%) and III (36.2%) periodontitis were the most prevalent, with PPD Mean ± SD 4.95 ± 1.38, CAL Mean ± SD 4.21 ± 1.38. The key risk factors for severe periodontitis included being male, being older, being less educated, being a smoker, and having diabetes. The study also revealed that irregular tooth brushing, residence in specific urban locations, and advanced periodontitis stages were significantly associated with poorer OHRQOL.

**Conclusions:**

Periodontitis adversely affects OHRQOL among Egyptian elderly individuals, with the psychological and physical domains being the most affected. These findings underscore the need for targeted public health strategies and personalized interventions to mitigate the burden of periodontal disease in this population.

## Introduction

Periodontal disease is an oral infection characterized by an imbalance between the host's immune response and bacterial challenges [1]. It encompasses a range of inflammatory conditions that lead to the degeneration of the periodontium, affecting structures such as the gingiva, periodontal ligament, cementum, and alveolar bone, often resulting in tooth loss. According to the World Health Organization (WHO) reports that approximately 10–15% of the global population suffers from severe periodontal conditions [2]. Significant correlations between periodontal disease and various systemic medical conditions have been identified in recent decades. Although definitive causal relationships have not been fully established, numerous studies highlight both positive and negative associations [3]. Globally, there is a notable demographic shift characterized by an aging population. In 2010, approximately 524 million individuals were aged 65 and older, with projections suggesting this number will rise to 1.5 billion by 2050. This trend highlights a growing population of older adults surpassing the number of young children for the first time in history [4]. According to the United Nations, the proportion of individuals aged 65 and above increased from 6% in 1990 to 9% in 2019, with projections indicating a further rise to 16% by 2050 [5]. This demographic shift necessitates a thorough scholarly investigation to understand its implications for social, economic, and healthcare systems. Despite advancements in understanding aging-related disorders, a gap remains in the literature regarding their organizational, a social, and economic impacts, particularly within the healthcare systems of low- and middle-income countries (LMICs) in the Arab world [6]. Periodontal disease significantly impacts an individual's overall health and quality of life, with prevalence increasing with age. In Western industrialized countries, the elderly population is expanding, and more individuals are choosing to retain their natural teeth, suggesting an expected rise in the incidence of periodontal disease [7]. Life expectancy in Egypt has significantly improved from 1980 to 2020, with projections indicating further increases by 2050. By then, Egypt's population is anticipated to reach 160 million, with a substantial proportion aged 60 or older. Historically labeled as a ‘young country,’ Egypt is expected to see an increase in its mean age, aligning more closely with demographic trends observed in European nations (Central Agency for Public Mobilization and Statistics, 2023) [6, 8]. Among the geriatric population, periodontal disorders are prevalent, with incidence and severity escalating due to progressive tissue degeneration associated with aging. If gingivitis remains untreated, periodontitis can exacerbate in advanced age. An alternative hypothesis for the correlation between periodontal tissue degeneration and aging is the increased vulnerability inherent in the aging process. Older adults are more prone to developing periodontal disease due to alterations in the immune response and prolonged exposure to risk factors. This heightened susceptibility is associated with biological aging, which impairs the body's capacity to maintain periodontal health [9]. Men and individuals with uncontrolled diabetes face an increased risk of periodontal disease as they age, a risk further compounded by smoking and inadequate oral hygiene [10]. Maintaining good oral health is critical for overall well-being. The World Health Organization (WHO) defines health as a state of complete physical, mental, and social well-being, rather than merely the absence of disease. Through its Global Oral Health Program, the WHO seeks to enhance awareness of oral health worldwide. Despite advancements, oral diseases persist as a significant health challenge, especially in less affluent countries. With the aging global population, there is an urgent need for research and enhanced public health strategies to address the evolving demands of oral health [11]. Oral diseases substantially affect an individual's quality of life due to their psychological and social repercussions.

Any medical condition that impedes daily activities can adversely affect overall quality of life. Consequently, oral health-related quality of life (OHRQOL) assesses the varied impacts of dental disorders on different life aspects [12]. Oral Health-Related Quality of Life (OHRQOL) is a multidimensional construct that subjectively evaluates functional and emotional well-being, as well as expectations and satisfaction associated with oral and dental health [13]. This assessment is conducted using indicators specific to oral health-related quality of life. The most commonly employed tool for evaluating OHRQOL is the Oral Health Impact Profile (OHIP) and its abbreviated form, OHIP-14, which includes 14 questions. OHIP-14 specifically measures the negative impacts of oral and dental issues, such as periodontal diseases, that compromise oral health [14, 15]. Oral Health-Related Quality of Life (OHRQOL) evaluates the extent to which oral health affects daily life, well-being, and overall satisfaction, taking into account factors such as function, psychological state, social interactions, and pain. Research indicates that poor oral health, particularly periodontal disease, significantly diminishes OHRQOL, especially among older adults who frequently encounter additional health challenges and diminished physical capacity. Therefore, understanding the impact of periodontal disease on the OHRQOL of older Egyptians is essential for developing targeted interventions aimed at enhancing the quality of life within this vulnerable population [16]. Recent studies have demonstrated a significant prevalence of periodontal disease among Egyptians, with marked variations across different ages and regions. A cross-sectional national survey underscored the widespread occurrence of periodontal issues among Egyptian adults, pinpointing critical risk factors such as inadequate oral hygiene, tobacco use, and systemic conditions like diabetes [13].

Elderly Egyptians are particularly susceptible to factors such as limited access to dental care, socioeconomic challenges, and a lack of oral health awareness. Research suggests that older individuals often prioritize systemic health over dental health, potentially exacerbating periodontal issues and affecting their overall quality of life [17]. Additionally, other common oral health problems, such as tooth loss, dental caries, and dry mouth, further contribute to the decline in oral health-related quality of life (OHRQOL) among elderly Egyptians [18]. Considering the substantial burden of periodontal disease and its impact on quality of life, this study aims to investigate the specific effects of periodontal disease on oral health-related quality of life (OHRQOL) among Egypt's elderly population. By analyzing the prevalence, severity, and risk factors associated with periodontal disease in this demographic, the study seeks to offer insights that could inform and improve public health policies and clinical practices. The ultimate objective is to enhance oral health outcomes and overall well-being among Egypt's aging population.

## Materials and methods

### Study design, settings, and participants

In this cross-sectional study, a random sample of 400 Egyptian geriatric patients aged over 60 years, frequently visited four different dental centers as an outpatient clinic, the periodontology department of Ain Shams University, Cairo Governorate**,** and the research centers of the Ministry of Health and Population, MOHP. The Dentists Training Center in Mansoura, Dakahlia Governorate. The Dentists Training Center in Damnhour, Beheira Governorate**.** The Specialized Dental Center in Port Said**,** Port Said Governorate. These centers were chosen based on their high geriatric patient flow and geographic distribution to capture diverse socioeconomic and health backgrounds. A random sampling technique was used within these centers to ensure that participants were not selectively chosen based on their oral health status, increasing the study's generalizability. Conducted in a single visit, thereby eliminating the need for follow-up assessments. Importantly, this specific dental clinic houses a removable dental laboratory, such a facility attracts a high flow of geriatric patients, who are the primary focus of our study, As a result, the sample although not fully representative of the general Egyptian elderly population, is still relevant to the study objective, as it captures a population segment closely aligned with the study focus on geriatric oral health and the impact on oral health-related quality of life. Inclusion/Exclusion Criteria: Inclusion: both genders, male and female patients aged ≥ 60 years diagnosed with periodontitis and willing to participate. Exclusion: Individuals with < 2 remaining teeth, severe cognitive impairments, or severe systemic diseases that might interfere with oral health assessment, direct socioeconomic exclusion criteria were not applied. The research adhered to established ethical guidelines, and the study was approved by the Faculty of Dentistry Ain Shams University Research Ethics Committee (FDASU-REC) with approval number (FDASU-REC IM052302) and the research ethical committee of the Egyptian Ministry of Health and Population, MOHP with approval number (12–2023/16). A participant’s confidentiality was protected, and their data were fully secured following ethical guidelines.

Written informed consent was signed by all participants prior to the clinical examination. In the case of illiterate patients, the consent form was read aloud to the participants. Their full understanding of the study details was ensured, and verbal consent was obtained in the presence of a witness, who then signed the consent form on behalf of the participant. Alternatively, the participants could provide consent by placing a thumbprint on the consent form.

### Questionnaire

All the participants answered a structured questionnaire before the clinical examination, The questionnaire contained items concerning a sociodemographic background, educational level, smoking habits, medical conditions, medication use, frequency of dental visits, and their regularity, and patterns of teeth brushing. Education levels were assessed by six alternative responses due to the variation in educational levels across the Egyptian population: “illiterate”, “primary”, “preparatory”, secondary”, and “university”. Additionally, smoking habits were assessed by three alternative responses: never smoker”, former smoker, and current smoker. The “Current smoker” was defined as an individual who smoked at least one cigarette daily. Current smokers also reported the number of cigarettes consumed daily. The oral health-related quality of life (OHRQOL) was assessed via the oral health impact profile (OHIP-14) [19, 20]. The questionnaire consists of 14 items divided into seven dimensions: functional limitation, physical pain, psychological discomfort, physical disability, psychological disability, social disability, and handicap.

Ratings are made on a 5-point Likert scale for each item: 0 = never, 1 = hardly ever, 2 = occasionally,

3 = fairly often, and 4 = very often, with the sum of the scores ranging from 0–56. Higher OHIP-14 scores indicate poorer OHRQOL. Participants reporting a negative impact (response codes: 3’fairly often’ and 4’very often’) on one or more of the 14 items were categorized as having a negative impact on OHRQOL, whereas those who had response codes ranging from 0–2 for all items were considered to have fair OHRQOL by the OHIP-14. Language and Cross-Cultural Adaptation of OHIP-14: The OHIP-14 questionnaire was used in Modern Standard Arabic, the primary language of participants. It has been previously validated for use in Arabic-speaking populations. No major cultural adaptations were needed, as the questionnaire's constructs align with standard oral health assessment metrics [21, 22].

### Clinical examinations

Two calibrated periodontists conducted clinical examinations. Inter-examiner reliability was tested using Cohen’s kappa (κ = 0.86), indicating high agreement. The clinical examination was divided into three segments the 1 st segment included recording the prosthesis if available as a removable partial or complete denture, and was fixed as a crown, bridge, or implants; the 2nd segment included registration of the DMF, and the total number of permanent teeth filled, missing, and decaying. Every tooth, excluding the third molars, was included [23, 24]. The 3rd segment included the recording of periodontal parameters such as tooth mobility, periodontal probing pocket depth (PPD), clinical attachment level (CAL), bleeding on probing (BOP), and the plaque index (PI). All periodontal parameters were recorded and written on periodontal charts [25].

Periodontal status was assessed based on the consensus report from the 2017 World Workshop on the Classification of Periodontal and Peri-implant Diseases and Conditions (2018 EFP/AAP classification) [26]. Plaque index (PI): Supragingival plaque presence was recorded on four tooth surfaces using this index. Plaque was documented as present (+) or absent (−), and the plaque incidence was calculated as a percentage [26]. Gingivitis Index (Bleeding on Probing (BOP): Gingival bleeding was assessed on all tooth surfaces, with bleeding recorded as either present (+) or absent (-). Gingivitis severity was expressed as a percentage [27, 28]. Clinical Attachment Loss (CAL): Indicates junctional epithelium migration and connective tissue loss, key in periodontitis assessment. Measured from the cementoenamel junction (CEJ) to the periodontal pocket base. CAL Calculations: Gingival margin at CEJ → CAL = PD, Coronal to CEJ → CAL = PD – GML, Apical to CEJ → CAL = PD + GML. CAL was recorded as a means of patient periodontal status. Periodontal Pocket Depth: Using a calibrated probe, measure six sites per tooth to assess periodontal health, PPD recorded as the mean of the patient's status. Periodontal health was comprehensively assessed by measuring probing depths and clinical attachment loss (CAL) at six sites per tooth. These individual measurements were summed to calculate the total probing depth and total CAL for each patient. The mean values were then determined by dividing these totals by the number of sites measured [29, 30]. Periodontitis is divided into four stages based on clinical attachment loss (CAL), bone loss, and probing pocket depth (PPD). Stage I has CAL of 1–2 mm and bone loss under 15%. Stage II shows CAL of 3–4 mm and bone loss of 15–33%. Stage III and IV involve CAL of 5 mm or more, bone loss to the middle root, and PPD of 6 mm or more, with Stage IV requiring complex treatment due to the loss of five or more teeth [31].

### Variables

Outcome (Dependent Variable): The primary focus of this study is the impact on Oral Health-Related Quality of Life (OHRQOL).

Main Exposure Variables (Key Predictors of OHRQOL): Periodontitis is assessed through periodontal parameters, including probing pocket depth (PPD), clinical attachment loss (CAL), and bleeding on probing (BOP).

Mediators (Pathways through which exposures affect OHRQOL): The Decayed, Missing, and Filled Teeth (DMF) index acts as a mediator, influencing the functional and aesthetic dimensions of quality of life. Additionally, the presence of dental prostheses affects functional capacity, aesthetic considerations, and adaptation to missing teeth.

Confounders (Factors influencing both exposure and outcome, potentially biasing the effect estimate): Age serves as a significant confounder, impacting both periodontal health and OHRQOL. Gender affects health behaviors and perceptions of quality of life, while educational level influences awareness of oral hygiene and access to dental care. Medical conditions such as diabetes and cardiovascular diseases also affect periodontal health and overall well-being. Smoking is a strong confounder, affecting both periodontal and systemic health.

**Fig. 1:**
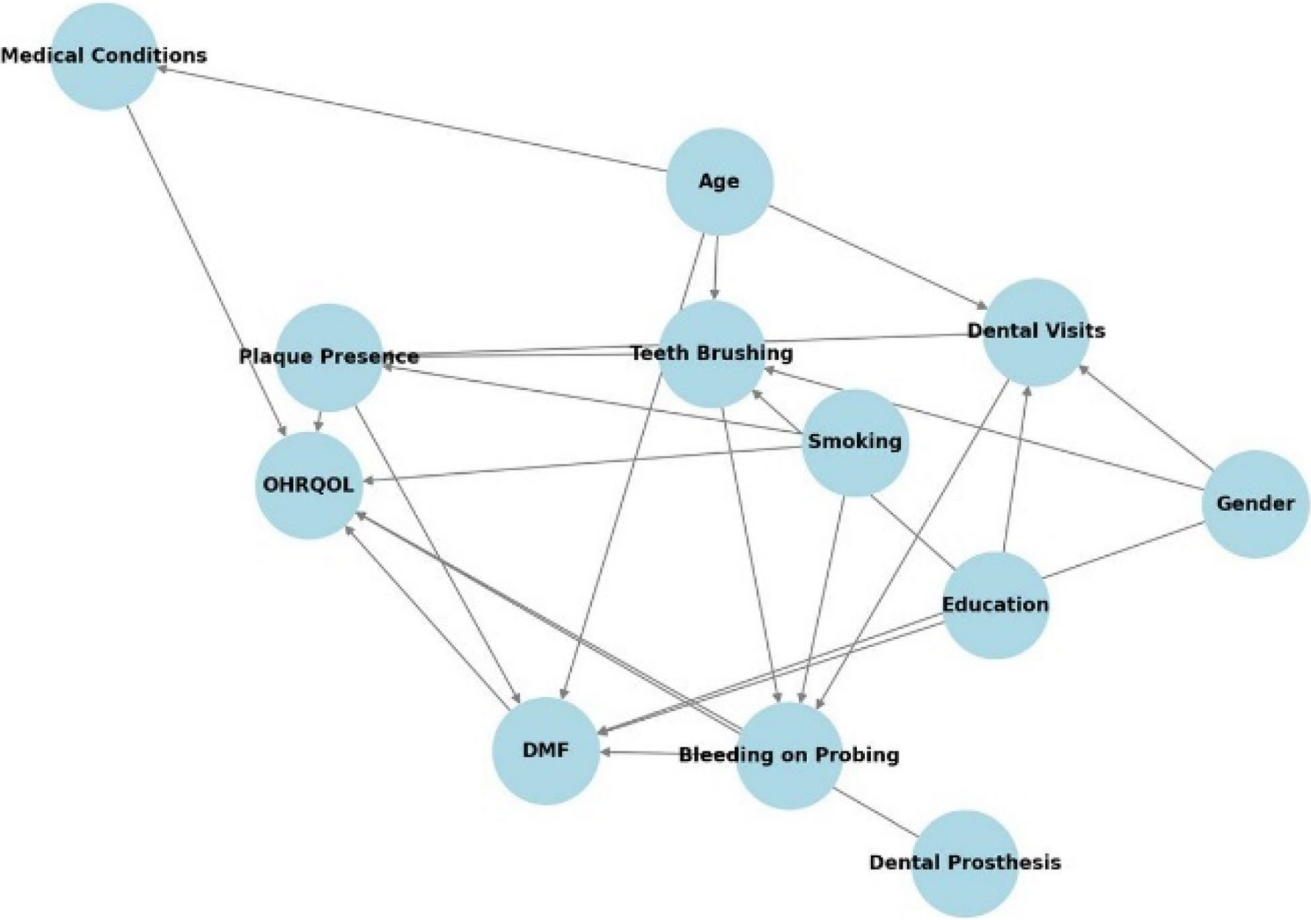
Directed Acyclic Graph (DAG) representing the relationships among periodontitis risk factors and OHRQOL

Colliders (Variables influenced by two or more other variables, introducing potential bias if adjusted for improperly): Variables such as tooth brushing and dental visits are influenced by education level and affect plaque presence and periodontal status. These variables must be carefully considered to avoid introducing bias in the analysis (Fig. 1).

### Study size and statistical analyses

The sample size was determined using G*Power software based on previous epidemiological data on periodontitis prevalence in geriatric populations (effect size = 0.3, power = 80%, α = 0.05). A random sampling approach was used within each center to minimize selection bias. Assuming a confidence limit of 5% and a confidence level of 95%, the minimum required sample size was 345 elderly patients. An additional 10% was added to compensate for potential losses, resulting in a total sample size of 400 cases after rounding. The collected data were analyzed using SPSS (Statistical Package for the Social Sciences), version 22. Quantitative data were tested for normality using the Kolmogorov‒Smirnov test and were then described as means and standard deviations for normally distributed data, and as medians and ranges for non-normally distributed data. Qualitative data were presented as numbers and percentages. The Chi-square test was used to examine associations between categorical variables (e.g., gender, age, education level) and their relationship with oral health-related quality of life (OHRQL). The Chi-square test also used to assess the stage of periodontitis in relation to oral health-related quality of life (OHRQOL). Logistic regression analysis was employed to evaluate the association between periodontitis severity and OHRQOL impairment, adjusting for potential confounders such as age, gender, and smoking habits. The results of the regression analyses were presented as odds ratios (ORs) with 95% confidence intervals (CIs). The level of significance was set at *p* < 0.05. The Kolmogorov–Smirnov test was used to assess normality prior to applying statistical tests.

## Results

### Patient characteristics

This cross-sectional study was conducted in a single visit, ensuring there was no participant attrition due to follow-up requirements. The study involved 400 participants, all aged 60 and above, consisting of 228 males (57%) and 172 females (43%). Among them, 169 participants (42.3%) were aged between 66 and 70 years. 141 participants (35.3%) had no formal education. Stage II periodontitis was present in 160 participants (40% of the sample), while Stage III periodontitis was observed in 145 participants (36.2%). With PPD Mean ± SD 4.95 ± 1.38, CAL Mean ± SD4.21 ± 1.38. Sociodemographic characteristics of the study population in relation to periodontitis stage shown in Table 1.

**Table 1:** Distribution of the studied samples according to periodontitis stage, according to their sociodemographic characteristics

Periodontitis stage					
	Total n (%)	Stage 1 n (%)	Stage 2 n (%)	Stage 3 n (%)	Stage 4 n (%)
Total	400 (100)	63 (15.8)	160 (40)	145 (36.2)	32 (8)
**Gender**					
Male	228 (57)	48 (76.2)	87 (54.4)	77 (53.1)	16 (50)
Female	172 (43)	15 (23.8)	73 (45.6)	68 (46.9)	16 (50)
**Settings**					
ASU	100 (25)	6 (9.5)	34 (21.3)	45 (31)	15 (46.9)
Mansoura	100 (25)	8 (12.7)	47 (29.4)	40 (27.6)	5 (15.6)
Port Said	100 (25)	22 (34.9)	44 (27.5)	31 (21.4)	3 (9.4)
Damanhour	100 (25)	27 (42.9)	35 (21.9)	29 (20)	9 (28.1)
**Educational level**					
Illiterate	141 (35.3)	16 (25.4)	51 (31.9)	60 (41.4)	14 (43.8)
Primary	59 (14.7)	8 (12.7)	28 (17.5)	19 (13.1)	4 (12.5)
Preparatory	49 (12.2)	8 (12.7)	22 (13.8)	16 (11)	3 (9.4)
Secondary	76 (19)	14 (22.2)	32 (20)	26 (17.9)	4 (12.5)
University	75 (18.8)	17 (27)	27 (16.9)	24 (16.6)	7 (21.9)
**Smoking**					
Never	270 (67.5)	47 (74.6)	114 (71.3)	89 (61.4)	20 (62.5)
Former	45 (11.2)	9 (14.3)	16 (10)	19 (13.1)	1 (3.1)
Current	85 (21.3)	7 (11.1)	30 (18.8)	37 (25.5)	11 (34.4)
**Medical condition**					
No	166 (41.5)	30 (47.6)	77 (48.1)	49 (33.8)	10 (31.3)
DM only	35 (8.8)	3 (4.8)	21 (13.1)	11 (7.6)	0 (0)
HTN only	52 (13)	13 (20.6)	17 (10.6)	20 (13.8)	2 (6.3)
Cardiac disease only	15 (3.8)	6 (9.5)	5 (3.1)	4 (2.8)	0 (0)
Bone disease	14 (3.5)	0 (0)	0 (0)	7 (4.8)	7 (21.9)
DM & HTN	43 (10.8)	5 (7.9)	16 (10)	19 (13.1)	3 (9.4)
DM, HTN & Cardiac disease	19 (4.8)	2 (3.2)	5 (3.1)	8 (5.5)	4 (12.5)
**Teeth brushing**					
None	175 (43.75)	22 (34.9)	65 (40.6)	72 (49.7)	16 (50)
Regular	95 (23.75)	25 (39.7)	33 (20.6)	33 (22.8)	4 (12.5)
Frequent/Irregular	130 (32.5)	16 (25.4)	62 (38.8)	40 (27.6)	12 (37.5)
**Dental visit**					
None	277 (69.3)	34 (54)	109 (68.1)	110 (75.9)	24 (75)
Regular	44 (11)	14 (22.2)	15 (9.4)	13 (9)	2 (6.3)
Irregular	79 (19.7)	15 (23.8)	36 (22.5)	22 (15.2)	6 (18.8)

*Abbreviation: DM* Diabetes Mellitus, *HTN* Hypertension

#### OHIP questionnaire

In this study, the Oral Health-Related Quality of Life (OHRQOL) was assessed using the abbreviated Norwegian version of the Oral Health Impact Profile (OHIP-14). Participants responded to each item using a 5-point Likert scale, with options ranging from 0 (never) to 4 (very often). The findings showed that 266 out of 400 participants, or 66.5%, experienced a negative impact, indicating poor oral health-related quality of life. OHRQOL outcome and statistical summary of its score shown in Table 2.

**Table 2:** Distribution of the studied sample according to the outcome OHRQOL- impact and summary statistics of its score

Items	N	%
OHRQOL impact		
Negative impact	266	66.5
Positive impact	134	33.5
Total	400	100
OHRQOL impact		
Mean ± SD		23.3 ± 7.8
Median (IQR)		23 (17,29)
Range (Min–Max)		Jan-43

*Abbreviation: OHRQOL* Oral Health-Related Quality of Life

### Periodontitis stage in relation to OHRQOL impact

**Fig. 2:**
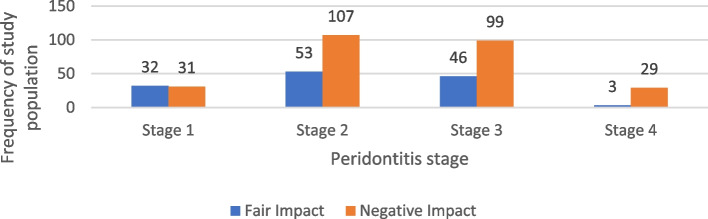
Frequency of the periodontitis stage of the study population according to their OHRQOL score

Our findings suggest that Stage II and Stage III periodontitis were the most frequently observed stages in our sample. Among the 266 individuals who reported a negative impact on Oral Health-Related Quality of Life (OHRQOL), 40.2% (107 participants) had Stage II periodontitis, while 99 individuals had Stage III. Similarly, among those who reported a fair impact, Stage II was the most common (39.6%, 53 participants), followed by Stage III (34.3%, 46 participants). Statistical analysis indicated a significant association between periodontitis stage and OHRQOL impact (*p* = 0.001), suggesting that individuals with more advanced periodontitis may be more likely to experience a greater negative impact on their OHRQOL. the impact of periodontists’ stage on OHRQOL among the study population shown in Tables 3. Table 4 presents the summary statistics of OHRQOL domain scores in relation to periodontitis stage. Figure 2 illustrates the distribution of periodontitis stages among the study population according to their OHRQOL scores.

**Table 3:** Distribution of the studied samples according to periodontitis stage in relation to OHRQOL impact

Variables	OHRQOL Impact				χ^2^	*P* value
	Negative impact *N* = 266		Fair impact *N* = 134			
	N	%	N	%		
Periodontitis stage						
Stage I	31	11.7	32	23.9		
Stage II	107	40.2	53	39.6	17.033	0.001*
Stage III	99	37.2	46	34.3		
Stage IV	29	10.9	3	2.2		

*Abbreviation: OHRQOL* Oral health-related quality of life, *χ*^*2*^ Chi-square test

**Table 4:** Summary statistics of OHRQOL domain scores of the studied sample regarding periodontitis stage

**OHRQOL** **Domains**	**Periodontitis stage**							
	Stage 1 *N* = 63		Stage 2 *N* = 160		Stage 3 *N* = 145		Stage 4 *N* = 32	
	Mean ± SD	Median (IQR)	Mean ± SD	Median (IQR)	Mean ± SD	Median (IQR)	Mean ± SD	Median (IQR)
**Functional limitation**	2.6 ± 1.6	3 (1,4)	2.7 ± 1.8	3 (1,4)	2.7 ± 1.7	3 (1,4)	3.3 ± 1.9	4 (1,5)
**Physical pain**	4.2 ± 1.3	4 (4,5)	4.6 ± 1.4	5 (4,6)	4.5 ± 1.6	5 (3,6)	5 ± 1.1	5 (4,6)
**Psychological discomfort**	3.5 ± 1.3	4 (2,4)	3.7 ± 1.4	4 (3,4)	3.6 ± 1.5	4 (3,4)	3.8 ± 1.1	4 (3,5)
**Physical disability**	3.4 ± 1.5	4 (3,5)	3.9 ± 1.7	4 (3,5)	3.8 ± 1.8	4 (2,5)	4.3 ± 1.4	4 (3,5)
**Psychological disability**	3 ± 1.5	3 (2,4)	3.4 ± 1.5	4 (2,4)	3.4 ± 1.5	4 (2,4)	4 ± 1.1	4 (3,5)
**Social disability**	2.4 ± 1.5	2 (1,4)	2.7 ± 1.4	3 (2,4)	2.9 ± 1.5	3 (2,4)	3.4 ± 1.6	3 (2,4)
**Handicap**	2.3 ± 1.7	2 (1,4)	2.4 ± 1.6	2 (1,4)	2.5 ± 1.6	2 (1,4)	3.2 ± 1.6	3 (2,4)

*Abbreviation: OHRQOL* Oral Health-Related Quality of Life

### The frequency percentage of the highest responses on the OHRQOL items

Analysis of the responses revealed that psychological discomfort (Domain 3) had the highest frequency, ranking first. This was followed by psychological disability (Domain 5) in second place, and physical pain (Domain 2) in third place. Frequencies and percentages of the highest scores on the ORHQOL items are shown in Table 5.

**Table 5:** Frequencies and percentages of the highest scores on the ORHQOL items

Variables	N	%
Domain 1: Functional limitation		
1. Have you had trouble pronouncing any words because of problems with your teeth, mouth, or dentures?		
Never	122	30.5
2. Have you felt that your sense of taste has worsened because of problems with your teeth, mouth, or dentures?		
Occasionally	157	39.3
Domain 2: Physical pain		
3. Have you had painful aching in your mouth?		
Occasionally	188	47
4. Have you found it uncomfortable to eat any foods because of problems with your teeth, mouth, or dentures?		
Occasionally	167	41.8
Domain 3: Psychological discomfort		
5. Have you been self-conscious because of your teeth, mouth, or dentures?		
Occasionally	208	52
6. Have you felt tense because of problems with your teeth, mouth, or dentures?		
Occasionally	187	46.8
Domain 4: Physical disability		
7. Has your diet been unsatisfactory because of problems with your teeth, mouth or dentures?		
Occasionally	168	42
8. Have you had to interrupt meals because of problems with your teeth, mouth or dentures?		
Occasionally	171	42.8
Domain 5: Psychological Disability		
9. Have you found it difficult to relax because of problems with your teeth, mouth or dentures?		
Occasionally	201	50.2
10. Have you been a bit embarrassed because of problems with your teeth, mouth or dentures?		
Occasionally	169	42.3
Domain 6: Social disability		
11. Have you been a bit irritable with other people because of problems with your teeth, mouth or dentures?		
Hardly ever	163	40.8
12. Have you had difficulty doing your usual jobs because of problems with your teeth, mouth or dentures?		
Hardly ever	157	39.3
Domain 7: Handicap		
13. Have you felt that life in general was less satisfying because of problems with your teeth, mouth, or dentures?		
Hardly ever	143	35.8
14. Have you been totally unable to function because of problems with your teeth, mouth, or dentures?		
Hardly ever	159	39.8

**Fig. 3:**
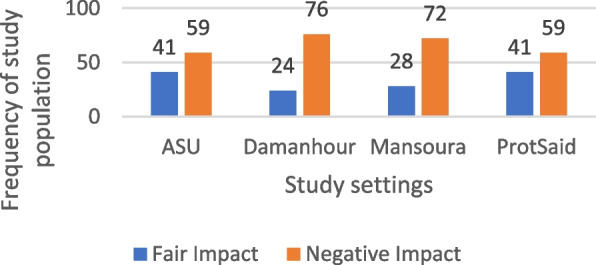
Frequency of study settings of the study population according to their OHRQOL impact

**Fig. 4:**
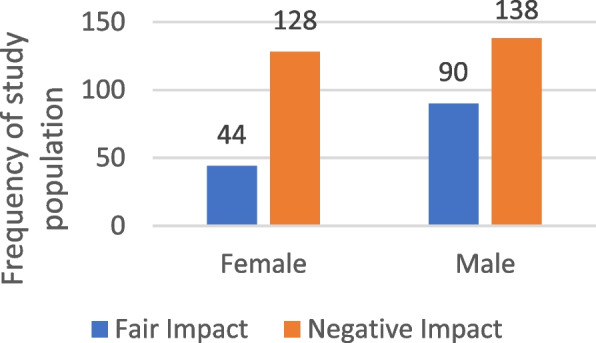
Frequency of the study population according to their OHRQOL impact

Our findings suggest that 66.5% of participants (266 out of 400) reported experiencing a negative impact on their Oral Health-Related Quality of Life (OHRQOL), while 33.5% (134 participants) reported a fair impact. The highest proportion of negative impact was observed in Damanhour at 28.6% (76 individuals). In contrast, Ain Shams and Port Said each had 30.6% (41 participants) reporting a fair impact. Statistical analysis indicated an association between study site and OHRQOL impact (*p* = 0.015). Gender analysis suggested a significant association, with 51.9% of males and 48.1% of females reporting a negative impact (*p* = 0.004). The 66–70 age group had the highest proportion of individuals reporting a negative impact (43.2%), though this association was not statistically significant. Among illiterate participants, 39.8% reported a negative impact, but this was also not statistically significant. That shown in Table 6, Figs. 3 and 4.

**Table 6:** Distribution of the studied sample according to their sociodemographic characteristics in relation to OHRQOL impact

Variables	QHRQOL Impact				χ^2^	*P* value
	Negative impact *N* = 266		Fair impact *N* = 134			
	N	%	N	%		
Study setting						
ASU (Ain-Shams University)	59	22.2	41	30.6	10.459	0.015*
Mansoura	72	27.1	28	20.9		
PortSaid	59	22.2	41	30.6		
Damanhour	76	28.6	24	17.9		
Gender						
Male	138	51.9	90	67.2	8.493	0.004*
Female	128	48.1	44	32.8		
Age Group						
60–65 years	51	19.2	34	25.4	3.628	0.459
66–70 years	115	43.2	54	40.3		
71–75 years	62	23.3	33	24.6		
76–80 years	24	9	7	5.2		
Over 80 years	14	5.3	6	4.5		
Educational level						
Illiterate	106	39.8	35	26.1	8.68	0.07
Primary	38	14.3	21	15.7		
Preparatory	31	11.7	18	13.4		
Secondary	43	16.2	33	24.6		
University	48	18	27	27		

*χ*^*2*^ Chi-square test

*Statistically significant at *P* value < 0.05

## Variables impacted on the OHRQOL

### DMF (decayed, missing, filled)

#### The average of DMF among the studied sample according to their QHRQOL Impact

**Fig. 5:**
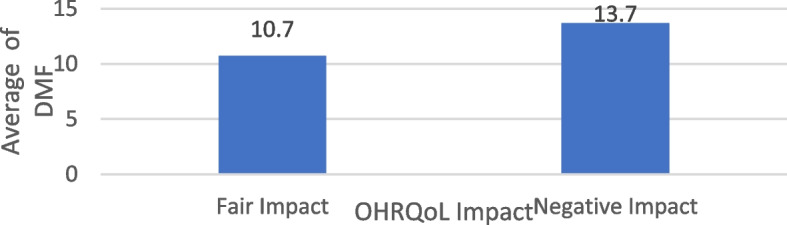
Differences in average DMF among the studied sample according to their OHRQL impact

This study explored the relationship between Oral Health-Related Quality of Life (OHRQOL) and Decayed, Missing, and Filled (DMF) scores among participants. Among individuals who reported experiencing a negative impact on their OHRQOL (*N* = 266), the average DMF score was 13.7 ± 4.4, with scores ranging from 2 to 25. In comparison, those who reported a moderate impact had an average DMF score of 10.7 ± 3.6, with a range of 0 to 21. Statistical analysis indicated a significant association between OHRQOL impact and DMF scores (*p* < 0.001), suggesting that individuals with greater OHRQOL impairment tended to have higher DMF scores. Differences in the average of DMF among the studied sample according to their OHRQOL Impact are shown in Table 7, Fig. 5.

**Table 7:** Differences in the average of DMF among the studied sample according to their OHRQOL Impact

Variables	OHRQOL Impact		U _*P*value_
	Negative impact *N* = 266	Fair impact *N* = 134	
DMF			
Mean ± SD	13.7 ± 4.4	10.7 ± 3.6	
Median (IQR)	13.5 (10,17)	10 (8,13)	< 0.001*
Range (Min–Max)	25-Feb	0–21	

*U* Mann Whiteny test

^*^Statistically significant at *P* value < 0.05

#### DMF average among the studied sample in relation to the periodontitis stage

**Fig. 6:**
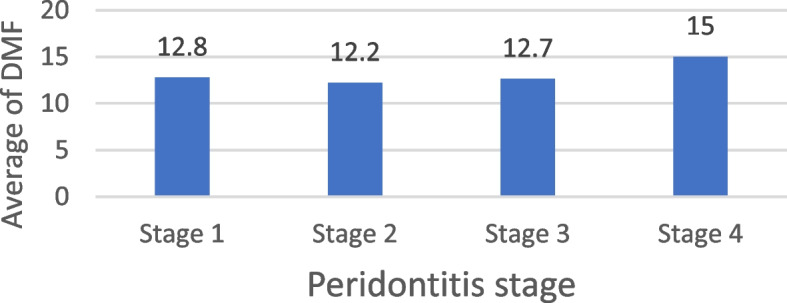
Differences in the average of DMF among the studied sample according to their periodontitis stage

The Decayed, Missing, and Filled (DMF) index may act as a mediator variable, potentially influencing the functional and esthetic impact on Oral Health-Related Quality of Life (OHRQOL). Statistical analysis suggested a significant association between the average DMF score and the stage of periodontitis (*p* = 0.013). Stages II and III of periodontitis were the most frequently observed among participants. Those with Stage II periodontitis had an average DMF score of 12.2 ± 4, with scores ranging from 2 to 23. Meanwhile, participants with Stage III periodontitis had an average DMF score of 12.7 ± 4.7, with scores ranging from 6 to 23. Differences in average DMF among the studied sample according to periodontitis stage Shown in Table 8, Fig. 6.

**Table 8:** Differences in average DMF among the studied sample according to periodontitis stage

Variable	DMF			*P* value
	Mean ± SD	Median (IQR)	Range (Min–Max)	
Periodontitis stage				
Stage I	12.8 ± 4.3	13 (9,16)	5–25	
Stage II	12.2 ± 4	12 (9,16)	2–23	0.013*
Stage III	12.7 ± 4.7	12 (9,16)	0–25	
Stage IV	15 ± 4.2	15.5 (12,18)	6–23	

Kruskal–Wallis test,

*Statistically significant at *P* value < 0.05

#### The removable and fixed prosthesis in relation to OHRQOL impact

**Fig. 7:**
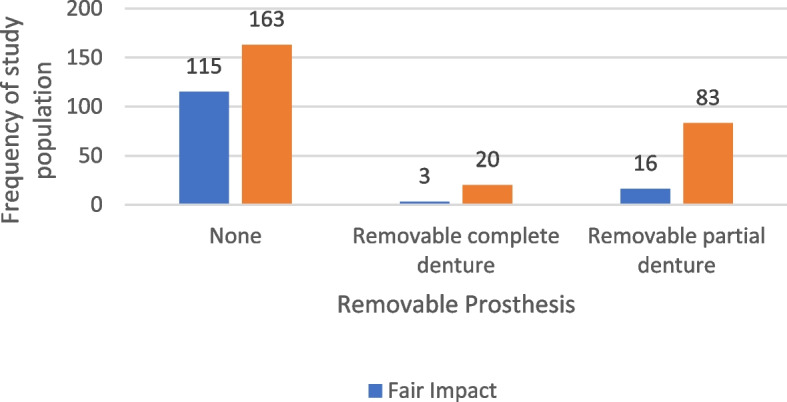
Frequency of type of removable prosthesis of the study population according to their OHRQOL impact

In our study, among participants who reported experiencing a negative impact on their Oral Health-Related Quality of Life (OHRQOL), 163 individuals (61.3%) did not have a removable prosthesis. In comparison, among those who reported only a fair impact, 115 out of 134 participants (85.8%) were without a removable prosthesis. Statistical analysis indicated a significant association between the presence of a dental prosthesis and OHRQOL (*p* < 0.001), suggesting that prosthesis use may be linked to variations in perceived oral health-related quality of life. The distribution of the studied sample according to the presence of removable & fixed prosthesis in relation to OHRQOL Impact is presented in Table 9, Fig. 7.

**Table 9:** The distribution of the studied sample according to the presence of removable & fixed prosthesis in relation to OHRQOL Impact

Variable	OHRQOL Impact				χ^2^	*P* value
	Negative impact *N* = 266		Fair impact *N* = 134			
	N	%	N	%		
Removable prosthesis						
None	163	61.3	115	85.8		
Removable partial denture	83	31.2	16	11.9	25.403	< 0.001*
Removable complete denture	20	7.5	3	2.2		
Fixed prosthesis						
None	169	63.5	77	57.5		
Crown	52	19.5	31	23.1	MC = 1.622	0.677
Bridge	40	15	24	17.9		
Crown and Bridge	5	1.9	2	1.5		

*χ*^*2*^ Chi-square test, *MC* Monte Carlo test when Chi-square test assumptions are violated

^*^Statistically significant at *P* value < 0.05

#### The bleeding on probing (gingivitis) and its presence in percentage in relation to the OHRQOL impact

**Fig. 8:**
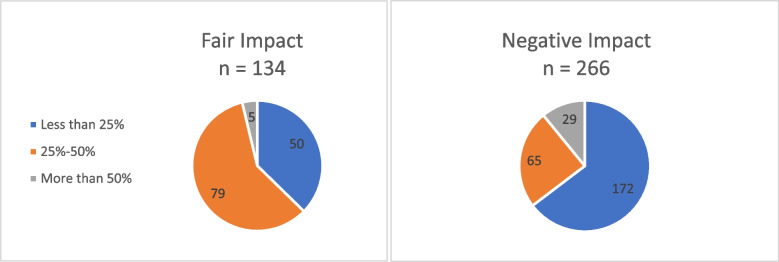
Frequency of the percentage of bleeding on probing of the study population according to their OHRQOL impact

All participants in the sample showed bleeding on probing, indicating gingivitis, affecting 100% of them. The extent of bleeding was most commonly observed within the 25–50% range. Out of 266 participants, 172 experienced a negative impact on Oral Health-Related Quality of Life (OHRQOL) and had bleeding on probing within this range. The findings suggested that gingivitis significantly impacts Oral Health-Related Quality of Life (OHRQOL) with a p-value of 0.004. The distribution of the studied sample according to bleeding on probing (gingivitis) and its percentage in relation to OHRQOL Impact shown in Table 10, Fig. 8.

**Table 10:** The distribution of the studied sample according to bleeding on probing (gingivitis) and its percentage in relation to OHRQOL Impact

Variable	OHRQOL Impact				χ^2^	*P* value
	Negative impact *N* = 266		Fair impact *N* = 134			
	N	%	N	%		
Bleeding on probing BOP						
Less than 25%	65	24.4	50	37.3	10.993	0.004*
25–50%	172	64.7	79	59		
More than 50%	29	10.9	5	3.7		

*χ*^*2*^ Chi-square test

*Statistically significant at *P* value < 0.05

#### Teeth brushing in relation to OHRQOL

**Fig. 9:**
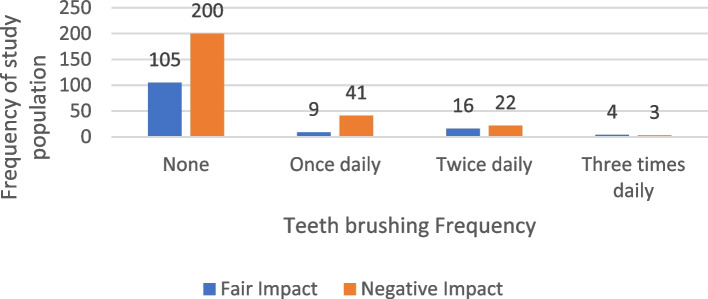
Frequency of teeth brushing frequency of the study population according to their OHRQOL impact

Among the 266 participants whose Oral Health-Related Quality of Life (OHRQOL) was negatively impacted, 118 individuals (44.4%) did not brush their teeth at all. In comparison, of the 134 participants reporting a fair impact on their OHRQOL, 57 individuals (42.5%) did not practice tooth brushing. Notably, even among those who brushed their teeth daily (*N* = 95), 62.1% still experienced negative impacts on their OHRQOL. The analysis indicates that tooth brushing significantly impacts Oral Health-Related Quality of Life (OHRQOL) with a *p*-value of 0.013. The distribution of the studied sample according to teeth brushing in relation to OHRQOL Impact is shown in Table 11, Table 12 displays the results of a multiple logistic regression analysis examining selected variables associated with the impact of the OHRQOL score. Table 13 presents a multiple logistic regression model evaluating the relationship between several independent variables and the stage of periodontitis. Table 14 presents the Logistic Regression of some independent variables in relation to the periodontitis stages. Figure 9 illustrates the frequency of tooth brushing among the study population according to their OHRQOL impact.

**Table 11:** The distribution of the studied sample according to teeth brushing in relation to OHRQOL Impact

Variables	OHRQOL Impact				χ^2^	*P* value
	Negative impact		Fair impact			
	N	%	N	%		
Teeth brushing						
None	118	44.4	57	42.5		
Regular	66	24.8	29	21.6	1.129	0.569
Frequent/Irregular	82	30.8	48	35.8		
Total	266	100	134	100		
Teeth brushing frequency among those brushing *N* = 95						
Once daily	41	62.1	9	31		
Twice daily	22	33.3	16	55.2	MC = 8.440	0.013*
3 times daily	3	4.5	4	13.8		
Total	66	100	29	100		

*χ*^*2*^ Chi-square test, *MC* Monte Carlo test when Chi-square test assumptions are violated

^*^Statistically significant at *P* value < 0.05

**Table 12:** Multiple logistic regression of some variables for the impact of the ORHQOL score

**Variables**	B coefficient	Odds ratio	*P* value	95% Confidence interval	
				Lower	Upper
Setting (Damanhour)**					
ASU (Ain-Shams University)	0.966	2.626	0.008*	1.289	5.351
Mansoura	0.056	1.058	0.878	0.515	2.172
PortSaid	0.531	1.700	0.135	0.847	3.410
Smoking (Former) **					
None	−0.704	0.495	0.020*	0.273	0.897
Smoker	−0.896	0.408	0.042*	0.172	0.968
Teeth brushing (Frequent/Irregular) **					
None	−0.362	0.696	0.190	0.405	1.197
Regular	−0.715	0.489	0.034*	0.253	0.946
Periodontitis stage (Stage IV) **					
Stage I	2.954	19.178	< 0.001*	4.503	81.672
Stage II	1.626	5.086	0.016*	1.347	19.207
Stage III	1.490	4.438	0.028*	1.178	16.712
DM	0.533	1.704	0.044*	1.014	2.866
DMF	−0.195	0.823	< 0.001*	0.772	0.878

*Abbreviation: DM* Diabetes Mellitus, *DMF* Decayed, Missed, Filled

^*^Statistically significant at *P* value < 0.05

^**^Considered constant

**Table 13:** Multiple logistic regression of several independent variables for the periodontitis stage

Variables	B coefficient	Odds ratio	*P* value	95% Confidence interval	
				Lower	Upper
Gender	1.244	3.468	< 0.001*	2.061	5.834
Age	0.979	2.663	< 0.001*	2.153	3.292
Educational level	−0.011	0.989	0.865	0.866	1.128
Smoking habit	1.007	2.738	< 0.001*	2.015	3.720
DM	0.587	1.798	0.006*	1.182	2.736
Hepatic disease	2.732	15.356	0.002*	2.811	83.875
Tumors	2.240	9.389	0.003*	2.155	40.915
Bone disease	3.435	31.019	< 0.001*	9.389	102.477

*Abbreviation: DM* Diabetes Mellitus

^*^Statistically significant at *P* value < 0.05

**Table 14:** Logistic Regression of some independent variables in relation to the periodontitis stages

Variables	B coefficient	Odds ratio	*P* value	95% Confidence interval	
				**Lower**	**Upper**
BOP	3.827	45.925	< 0.001*	2.562	5.092
DMF	0.054	1.055	0.351	−0.060	0.168
PD	3.047	21.052	< 0.001*	1.974	4.121
CAL	3.175	23.927	< 0.001*	2.295	4.056

^*^ Statistically significant at *P* value < 0.05

## Discussion

Our study aimed to investigate the impact of periodontitis and its risk factors on oral health-related quality of life OHRQOL among Egyptian geriatric patients. The results showed periodontitis adversely affects OHRQOL among Egyptian elderly with the psychological and physical domains being the most affected, and this aligned with studies from Western industrialized countries that report psychological discomfort and physical pain as key OHRQOL determinants in elderly patients with periodontitis [32]. In various studies, the perception of oral health impacts varies by region. A study conducted in Sri Lanka identified physical pain as the most prevalent complaint [14], while research from Germany highlighted functional limitation as the primary concern [33]. In contrast, psychological discomfort was most frequently reported in another study, whereas handicap was the least reported [34]. The study identifies Stage II and Stage III periodontitis as the most prevalent stages. Among 266 participants with negative Oral Health-Related Quality of Life (OHRQOL), 40.2% had Stage II, and a significant portion had Stage III periodontitis. The correlation between periodontitis stage and OHRQOL impact was statistically significant, with a p-value of 0.001. Another study found that the severity and progression of periodontitis were associated with poor oral health-related quality of life in patients with stage III-IV disease, and periodontitis had a greater impact than stage I-II periodontitis [32]. Compared to Stage III, Stage IV leads to more severe outcomes, such as complex tooth loss, significant chewing difficulties, and extensive bite collapse, all of which exacerbate the negative impact on OHRQOL [35]. These severe manifestations highlight the urgent need for early intervention and comprehensive management strategies for those at risk of advancing to this stage. Our research did not establish a definitive correlation between residency and the severity of periodontitis, Controversy other studies suggest that the risk of periodontitis may be influenced by residency, potentially due to factors such as language barriers, social deprivation, trauma, cultural differences, and unfamiliarity with the healthcare system [36]. Previous studies have noted that communication challenges between dentists and patients can hinder effective education on periodontal diseases and self-care [37, 38]; However, our study's setting yielded a statistically significant result, indicated by a p-value of 0.015, showing that residential location notably affects Oral Health-Related Quality of Life (OHRQOL). The Dentists Training Center in El-Mansoura exhibited the highest negative impact on OHRQOL, the Specialized Dental Center in Port Said, and Ain Shams University participants reported a fair impact on OHRQOL. The results showed an increased risk for severe periodontitis among smokers, individuals with diabetes type 2, and low educational level. Smoking has been extensively documented as a significant risk factor for periodontitis in prior research studies. Given that this risk factor is modifiable, enhancing public awareness and providing targeted information to patients with periodontitis could play a crucial role in decreasing its prevalence [39, 40]. Individuals with type 2 diabetes face a heightened risk of developing severe periodontitis, a finding that aligns with earlier research. This association underscores the critical need for diabetic patients and their healthcare providers to be well-informed about this association [40, 41]. The study did not establish a statistical significance between educational level and the severity of periodontitis, nor with oral health-related quality of life. This may be due to variations in oral hygiene behaviors across different educational backgrounds. Nevertheless, other studies have found that educational level influences oral conditions and should be considered when assessing risk and planning appropriate preventive measures [42]. Literacy and improvement of educational level could play in shaping oral health behaviors and Oral Health-Related Quality of Life (OHRQOL). Individuals with higher literacy are more likely to practice good oral hygiene, like regular toothbrushing and dental visits, while those with lower literacy levels often face increased plaque and periodontal disease. Limited health literacy can also hinder the understanding of oral health instructions, making it difficult for patients to recognize symptoms, seek timely care, and follow treatment recommendations [43]. This can worsen oral health conditions, causing pain and psychological distress, which negatively affect OHRQOL [44]. Other studies'results supported the efficacy of the educational intervention in enhancing knowledge, literacy, and behaviors associated with oral health and overall well-being [45]. Accordingly, previously published studies have shown that frequent tooth brushing is associated with improved oral health-related quality of life in geriatric individuals [46]. As we have reported in our study, regular tooth brushing has a statistically significant effect on oral health-related quality of life among a sample of geriatric individuals, with a p-value of 0.034 and an odds ratio of 0.489. Further research is warranted to explore the underlying mechanisms and develop effective interventions aimed at preventing the progression of periodontitis and alleviating its extensive burden on affected individuals.

## Study limitation

This study's cross-sectional design limits its ability to assess causality between exposure and outcome variables, for which longitudinal methods are preferred. Additionally, the low prevalence of stage IV periodontitis complicates analysis of its relationship with Oral Health-Related Quality of Life (OHRQOL). Increasing sample size and geographic diversity could improve generalizability and clarity of findings. Despite these constraints, the study offers a valuable base for future research, emphasizing the need for more targeted studies to explore the complex interactions between oral health and quality of life.

## Conclusions

This study examined the impact of periodontitis on the oral health-related quality of life (OHRQOL) of elderly individuals in Egypt, revealing significant negative effects, particularly in psychological and physical domains. These findings underscore the urgent need for public health strategies and personalized interventions to alleviate the burden of periodontal disease in this population.

Key factors associated with periodontitis severity included gender, age, education level, smoking status, and type 2 diabetes. Although lower education is typically linked to poorer oral health behaviors, our study found no direct statistical significance, suggesting that factors like smoking and diabetes might have a more profound impact.

Periodontitis significantly diminishes the quality of life for older adults in Egypt, especially regarding their psychological and physical well-being. This calls for targeted interventions and increased public awareness to enhance oral health in this demographic. Further research with larger populations is necessary to explore the relationship between periodontitis stage-grade and OHRQOL.

## Data Availability

The datasets used and/or analyzed during the current study are available from the corresponding author upon reasonable request.

## Abbreviations

AAP: American Academy of Periodontology
ASU: Ain Shams University
BOP: Bleeding on Probing
CAMPAS: Central Agency for Public Mobilization and Statistics
CAL: Clinical Attachment Level
CEJ: Cemento-enamel Junction
CI: Confidence Interval
DM: Diabetes Meletus
DMF: Decayed, Missed, Filled
EFP: European Federation of Periodontology
FDASU-REC: Faculty of Dentistry Ain-Shams University Research Ethics Committee
GBD: Global Burden of Diseases
HRQOL: Health-Related Quality of Life
HTN: Hypertension
LMICS: Low and Middle-Income Countries
NCD: Non-Communicable Diseases
NIH: National Institute of Health
OECD: Organization for Economic Cooperation and Development
OHRQOL.: Oral Health-Related Quality of Life
PI: Plaque Index
PPD: Probing Pocket Depth
QOL: Quality of Life
SUNT: Sound Untreated Natural Teeth
UN: United Nations
WHO: World Health Organization
YLD.: Years Lived with Disability
